# Apatinib is effective for treatment of advanced hepatocellular carcinoma

**DOI:** 10.18632/oncotarget.22337

**Published:** 2017-11-06

**Authors:** Yinlong Kong, Lin Sun, Zhenyu Hou, Yongqiang Zhang, Ping Chen, Yunlong Cui, Xiaolin Zhu, Tianqiang Song, Qiang Li, Huikai Li, Ti Zhang, Lunxiu Qin

**Affiliations:** ^1^ Tianjin Medical University Cancer Institute and Hospital, Key Laboratory of Cancer Prevention and Therapy, National Clinical Research Center for Cancer, Tianjin 300060, China; ^2^ Department of General Surgery, Huashan Hospital & Cancer Metastasis Institute, Fudan University, Shanghai 200040, China; ^3^ Cancer Research Center, Institute of Biomedical Science, Fudan University, Shanghai 200032, China

**Keywords:** apatinib, hepatocellular carcinoma, vascular endothelial growth factor receptor

## Abstract

As treatment options for hepatocellular carcinoma (HCC) are currently limited, we evaluated the efficacy and safety of oral apatinib, a VEGFR-2 inhibitor, on patients with advanced HCC. Twenty-two patients from Tianjin Medical University Cancer Institute and Hospital were enrolled for evaluation. Apatinib was administered at 500 mg/day or 250 mg/day continuously. Clinical endpoints were time to disease progression (TTP), overall survival (OS), and safety. The median TTP of treated patients was 10.4 months (95% CI 3.4 -17.5). At the last follow-up, 50% patients had survived longer than 11.4 months from the first dose. Complete response (CR), partial response (PR), stable disease (SD), and progressive disease (PD) rates were 0%, 40.9%, 40.9%, and 18.2%, respectively. The most common apatinib-related adverse events were hand-foot skin reaction (HFSR) (81.8%) and diarrhea (77.3%). Hypertension (27.3%) and HFSR (13.6%) were the most frequent grade 3/4 adverse events. In summary, results of this small study indicate that apatinib is well tolerated and extremely effective for the treatment of advanced HCC. It is therefore imperative to design and carry out well-controlled clinical trials to confirm its efficacy.

## INTRODUCTION

Hepatocellular carcinoma (HCC) is a major public health problem, ranking fourth in incidence and third in cancer-related mortality in China [1]. The main reason of HCC's high mortality rate is the lack of effective detection in the early stage. Consequently, it is estimated that 70%-85% of patients are diagnosed in the advanced stage [2]. For these patients, current treatment options show only modest efficacy.

Sorafenib is the standard treatment for advanced HCC. As a multi-target kinase inhibitor, sorafenib can suppress HCC cell proliferation by inhibiting the Raf/MEK/ERK signaling pathway. In addition, sorafenib blocks angiogenesis in HCC by inhibiting VEGFR and PDGFR [3]. The phase III SHARP [4] and ORIENTAL [5] clinical trials indicated that sorafenib prolongs overall survival in advanced HCC patients. However, the partial response rates of HCC to sorafenib were only 2% and 3.3%, and the extended survival was limited to 2.8 and 2.3 months, respectively. Furthermore, the occurrence of drug resistance and the expensive costs of treatment greatly limit the clinical application of sorafenib.

Many target drugs against advanced HCC continue to emerge from clinical trials, but none of them have so far proved to be superior to sorafenib. Recently, apatinib, an oral small molecule anti-angiogenesis agent, came to light due to its positive effect on advanced gastric cancer [6, 7] and HCC [8]. Apatinib exerts wide-ranging tumor inhibition both *in vitro* and *in vivo* by binding VEGFR-2 specifically [9, 10], and has been approved for the third-line treatment of gastric adenocarcinoma and gastroesophageal junction adenocarcinoma after phase II/III clinical trials [6, 7]. Moreover, numerous clinical studies of apatinib on solid tumors, such as non-small cell lung cancer, breast cancer, and HCC, have shown promising clinical efficacy [8, 11–14].

Considering the limitations of sorafenib, as well as the experience derived from a small group of advanced HCC patients treated with apatinib at our institute, we carried out a clinical study to explore the efficacy and safety of apatinib in advanced HCC.

## RESULTS

This study was carried out in patients who were sorafenib-resistant or could not afford the costs of sorafenib and were advised to receive apatinib. Written informed consent was obtained from 22 enrolled patients (19 males and 3 females), aged 32 to 77 years old (average, 54.3 years old). Among them, 21 patients had chronic hepatitis B virus infection and 13 had received antiviral therapy; 11 patients had postoperative recurrence and metastasis; 11 presented with macro-vascular invasion; 16 showed intrahepatic metastasis; 15 received previous or concomitant trans-arterial chemoembolization (TACE); and 21 had abnormally high serum alpha-fetoprotein (AFP) (Table 1).

**Table 1:** Univariate and multivariate analyses of time to disease progression in advance HCC patients under the treatment of apatinib

			Univariate analysis		Multivariate analysis	
Variable	N (%)	mTTP(month)	95%CI	p Value	Relative risk (95%CI)	p Value
Median age(Year)	54.3(32-77)			0.298		
<65	22	7.1	2.1-12.0			
≥65	4	10.6	——			
Gender				0.632		
Male	19	10.4	2.7-18.1			
Female	3	4.7	——			
ECOG- PS				0.944		
0	3	10.4	——			
1	18	7.0	2.4-11.7			
2	1	10.6	——			
HBs Ag				0.268		
(+)	21	10.4	4.7-10.9			
(−)	1	——	——			
Antivirus treatment				0.916		
No	8	10.6	10.3-10.9			
Yes	13	7.0	4.0-10.2			
Combined TACE				0.096		
NO	7	10.9	3.3-18.4			
Yes	15	7.0	0.2-17.5			
Relapse				0.910		
NO	11	10.9	0.4-21.4			
Yes	11	10.4	3.7-17.2			
PVTT				0.494		
NO	11	10.9	3.5-17.3			
Yes	11	10.4	5.1-16.7			
Metastasis				0.162		
NO	6	13.6	1.5-20.2			
Yes	16	10.4	3.4-17.5			
Child-Pugh				0.087		
A	17	7.1	3.6-10.5			
B	5	10.9	10.4-11.3			
ALT(U/L)	47.2(15.0-236.0)			0.641		
≥40	9	4.7	3.4-17.5			
<40	13	10.4	3.6-17.3			
AST(U/L)	64.2 (24.0-247.0)			0.707		
≥40	15	7.0	1.7-12.4			
<40	7	10.4	0.4-20.5			
ALP(U/L)	146.4 (25.0-293.0)			0.704		
≥125	13	10.6	0-22.0			
<125	9	7.1	4.6-9.6			
ALB(g/L)	38.0(26.0-53.1)			0.013		
≥40.0	10	5.8	2.3-9.4			
<40.0	12	10.9	10.4-11.3			
TBIL(μmol/L)	23.9(9.9-72.4)			0.031		
≥21.0	11	10.9	2.0-19.8			
<21.0	11	5.8	3.4-17.5			
DBIL(μmol/L)	7.4 (2.0-39.8)			0.838		
≥3.4	14	10.4	0-21.1			
<3.4	8	7.1	4.4-9.7			
HGB(g/L)	138.7 (87.0-185)			0.719		
≥130.0	16	7.1	3.6-10.5			
<130.0	6	10.6	——			
PLT(10^9^/L)	147.8 (40.0-410.0)			0.031	7.753 (1.160-51.823)	0.035
≥125	13	10.6	3.4-17.5			
<125	9	4.7	3.5-5.9			
AFP >ULN	21					
AFP reduction				0.012	0.117 (0.20-0.692)	0.018
AFP reduction (+)	14	10.6	2.6-18.6			
AFP reduction (−)	7	3.6	0.2-7.2			

ECOG PS=Eastern Cooperative Oncology Group Performance Status. BCLC=Barcelona Clinic Liver Cancer. HBV=hepatitis B virus. HCV=hepatitis C virus. TACE=trans arterial chemoembolization. PVTT=portal vein tumor thrombus. ULN, upper limit of normal.

**Figure 1:**
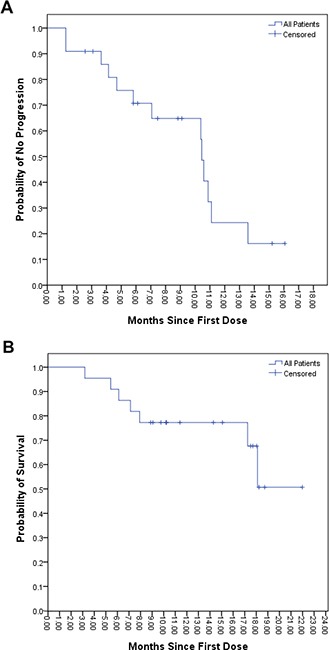
Kaplan–Meier Analysis of Time to Progression and Overall Survival For 22 HCC patients receiving apatinib, the median time to progression was 10.4 months (Panel **A**). The median overall survival is not available, because only 7 patients had died by the end of observation. However, half of the 22 patients survived more than 11.4 months at this time point (Panel **B**).

**Figure 2:**
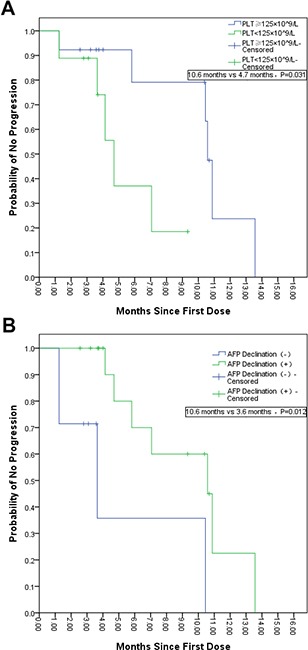
Kaplan–Meier Analysis of the Time to Progression of Selected Subgroups Multivariate analysis showed that platelet count and AFP reduction are associated with mTTP. The mTTP was only 4.7 months in patients with decreased platelet count (<125×10^9^/L), compared to 10.6 months in patients without decreased platelet count (≥125×10^9^/L) (Panel **A**). For patients with decreased AFP, mTTP was 10.6 months, compared to 3.6 months in patients without AFP response (Panel **B**).

**Figure 3:**
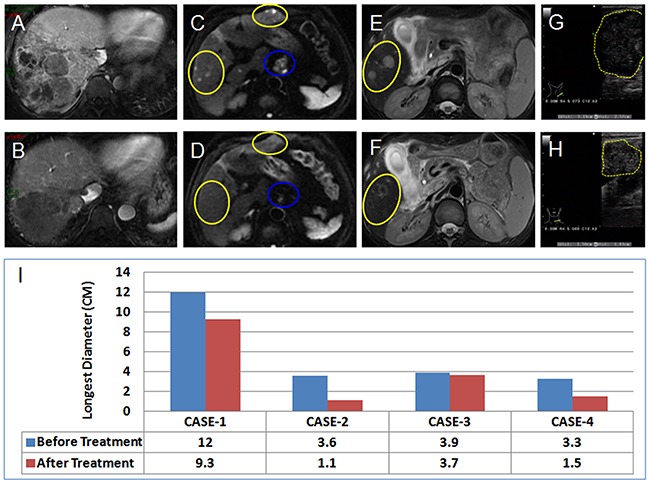
Typical Imaging Changes in Advanced HCC Patients Treated with Apatinib Tyical imaging presentations in 4 patients with advanced HCC (BCLC-C) before **(A, C, E, G)** and after **(B, D, F, H)** treatment with apatinib. (A and B) (Case 1), Portal-phase gadolinium-enhanced MR image showing that both a tumor in the liver and an embolus in the vena cava shrank significantly in a PR patient. (C and D) (Case 2), Diffusion-weighted MR imaging showing that intrahepatic metastatic HCC tumors (yellow-circled) disappeared after 4 months of treatment, while metastatic retroperitoneal lymph nodes (blue-circled) shrank dramatically in a PR patient. (E and F) (Case 3), T2-weighted MR image showing significantly decreased tumor activity (yellow-circled) in an SD patient. (G and H) (Case 4), B-ultrasound scan showing a significantly shrunken metastatic infraclavicular lymph node in a PR patient. **(I)** Changes in tumor size in the longest diameter before and after apatinib treatment (as shown above).

By the end of the last follow-up (July 20th, 2017), the median time to disease progression (mTTP) for these 22 patients was 10.4 months (95% CI 3.4-17.5). Seven patients died and 11 patients survived more than 11.4 months (Figure 1). Efficacy evaluation showed that complete response (CR), partial response (PR), stable disease (SD), and progressive disease (PD) were 0% (0/22), 40.9 % (9/22), 40.9 % (9/22) and 18.2% (4/22), respectively. The objective response rate (ORR) was 40.9% (9/22). Univariate analysis showed that the following factors may affect TTP: Albumin (ALB) ≥40.0 g/L (p = 0.013); Total Bilirubin (TBIL) <21.0 μmol/L (p = 0.031); Platelet (PLT) <125×10^9^/L (p = 0.031); and a decrease in AFP (p = 0.012). Multivariate analysis showed that TTP may be affected by: PLT <125×10^9^/L [HR 7.753 (95% CI 1.160-51.823); p = 0.03]; and a decrease in AFP [HR 0.117 (95% CI 0.30-0.692); p = 0.018] (Table 1 and Figure 2). After administration of apatinib, a reduction in AFP levels (i.e. AFP response) occurred in 14 cases, with 7 cases registering a reduction by half or more. Moreover, among these 7 cases, 6 were defined as PR with AFP reaching normal level in 2 cases. Typical imaging changes in patients with advanced HCC receiving apatinib treatment are shown in Figure 3 and in Supplementary Figures.

Adverse events in the 22 patients mainly consisted of hand-foot skin reaction (HFSR) (81.8%), diarrhea (77.3%), hypertension (63.6%), fatigue (59.1%), hoarseness (54.5%), and nausea (50%). Grade 3 or 4 drug-related adverse events mainly included hypertension (27.3%), HFSR (13.6%), and thrombocytopenia (9.1%). One case of upper gastrointestinal bleeding (in a patient receiving combined radiotherapy for bone metastasis) and 2 cases of cerebral hemorrhage were also recorded among patients with poor hypertension control. Because of hypertension, HFSR, diarrhea, and fatigue, 8 patients interrupted treatment, and another 6 required dose reduction. Meanwhile, 2 patients were withdrawn from treatment due to cerebral hemorrhage (Table 2).

**Table 2:** Apatinib treatment-related adverse events

Treatment-Related AEs	Any Grade No. (%) N=22	Grade 3 or 4 No. (%) N=22
Nonhematologic AEs		
hand-foot skin reaction	18 (81.8)	3 (13.6)
Diarrhea	17 (77.3)	1 (4.5)
Hypertension	14 (63.6)	6 (27.3)
Fatigue	13 (59.1)	0 (0)
Hoarseness	12 (54.5)	1 (4.5)
Rash	11 (50.0)	0 (0)
Decreased appetite	11 (50.0)	0 (0)
Vomiting	4 (18.2)	0 (0)
Proteinuria	4 (18.2)	1 (4.5)
Hematologic AEs		
Thrombocytopenia	14 (63.6)	2 (9.1)
Leukopenia	13 (59.1)	1 (4.5)
Neutropenia	12 (54.5)	1 (4.5)
Neutropenia	12 (54.5)	1 (4.5)
Anemia	7 (31.8)	0 (0)
Serum chemistry AEs		
Bilirubin increased	13 (59.1)	1(4.5)
ALT /AST increased	13 (59.1)	0 (0)

Abbreviations: AE, adverse event; ALT, alanine aminotransferase; AST, aspartate aminotransferase.

## DISCUSSION

Systemic treatment of advanced HCC remains a clinical challenge due to lack of effective drugs. Many first- and second-line agents have been tested in recent years, but the results were disappointing [15]. Currently, sorafenib is the only molecular targeted agent for advanced HCC that has received FDA approval after showing effectiveness in clinical trials. However, drug resistance and expensive treatment costs limit the application of sorafenib in the clinic.

The aim of the present study was to assess the efficacy and safety of daily administration of apatinib in the treatment of advanced HCC. In our patient cohort, mTTP (10.4 months) and ORR (40.9%) were much better than expected. As a matter of fact, results from the SHARP study (mTTP: 5.5 months; ORR: 2%) [4] and the ORIENTAL study (mTTP: 2.8 months; ORR: 3.3%) [5] lagged far behind our own. Because less than half of the patients died by the end of the last follow-up, this study failed to obtain mOS. However, half of the 22 patients evaluated survived over 11.4 months, an outcome also superior to that of the ORIENTAL study (mOS: 6.5 months).

In the present study, the first patient who received apatinib treatment was an elderly female who had been treated with sorafenib and regorafenib (clinical trial) because of metastasis and recurrence after HCC surgery. Drug resistance, multiple liver lesions, and multiple metastases to the abdominal cavity and lung developed in this patient during sorafenib and regorafenib treatment. This PR case attracted our attention because, surprisingly, her serum AFP level dropped from 899.2 μg/L to 74.03 μg/L after administration of apatinib for only 4 weeks. Moreover, 12 weeks later, not only her AFP values were within normal range, but also the tumors in her liver, abdominal cavity, and lung had shrank or even disappeared. Noteworthily, only 7/299, and 5/150 sorafenib-treated patients in the SHARP and the ORIENTAL studies, respectively, had PR. Although the present data are not directly comparable with these two clinical studies, the fact that 9 out of 22 apatinib-treated patients in our study showed PR is very impressive and extremely inspiring.

Preclinical animal experiments and clinical trials showed that apatinib-related adverse events were dose dependent [9, 16]. In this study, we mainly used apatinib at 250 mg/day, which is markedly lower than the previously tested doses of 750-850 mg/day [14]. In fact, patients who were initially treated at 500 mg/day suffered from serious adverse events and were switched to 250 mg/day. In general, patients receiving this low dose treatment had fewer and milder adverse events and achieved a good response. We speculate that this may be related to the background of liver disease in patients with HCC. Most patients with advanced HCC present also with chronic hepatitis or liver cirrhosis, and the poor hepatic functional reserve of these patients may affect the metabolism of apatinib. In addition, the general condition of patients with advanced HCC is not very good, which may further reduce the tolerance to apatinib-induced adverse events.

A phase II study showed that apatinib provides a potential survival benefit in HCC patients, and 850 mg/day or 750 mg/day were the recommended doses for further clinical studies [14]. In our study, however, significant success was achieved using a relatively low dose of apatinib. Moreover, we noticed that none of the 6 patients in this cohort initially treated with 500 mg/day apatinib could tolerate this dose for more than 3 months. Nevertheless, our study showed that grade 3 or 4 adverse events may occur with low-dose apatinib, similarly to what has been reported for this drug in other tumor therapies [10, 11, 17]. Unlike the sorafenib study, our data did not indicate a correlation between efficacy and adverse events of apatinib. However, due to the limited sample size, this is not a definitive conclusion.

Considering the potential benefits of interventional therapy in HCC treatment, we analyzed the efficacy of apatinib treatment alone or combined with interventional therapy. The results indicated that the effect of interventional therapy did not increase or decrease the efficacy of apatinib. Similarly, the SPACE study showed that sorafenib did not increase the efficacy of TACE in middle stage HCC patients [18]. It is worth noting that 4 patients in our study received interventional therapy as the first choice. Due to ineffectiveness of treatment (PD), these patients were then treated with apatinib. Among them, 3 achieved PR and one reached SD. These results further suggest that apatinib may be efficacious in the adjuvant setting.

AFP is considered an important marker in the diagnosis and treatment of HCC. Chan et al. [19] and Shao et al. [20] defined the AFP response as a 20% reduction in serum AFP levels after treatment. Data have shown that an AFP decrease is a good prognostic surrogate in patients with HCC after radiofrequency ablation [21] or sorafenib treatment [22], whereas an early increase in AFP predicted unfavorable clinical outcomes in patients with advanced HCC treated with sorafenib [23]. Our results suggest that a decrease in AFP serum levels may be helpful in judging the efficacy of apatinib in advanced HCC therapy.

In this study, thrombocytopenia was found to be another risk factor for TTP. Platelet count can reflect the severity of liver cirrhosis, and is associated with prognosis in HCC [5, 24, 25]. Therefore, it is not surprising that thrombocytopenia increased the overall and tumor-related risk of death in this cohort. Perhaps the fundamental cause of this phenomenon is the effect of cirrhosis on the efficacy of apatinib.

In conclusion, apatinib administration achieved an unprecedented therapeutic effect with an acceptable safety profile in patients with advanced HCC in this small sample size study. Although several limitations may somewhat compromise the reliability of our results (i.e. this being a single center retrospective study, its limited sample size, the potential benefits afforded by combined TACE therapy, and our inability to register mOS), the high PR rate and long mTTP obtained are very impressive. In light of the present results, we believe that it is imperative to design and carry out well-controlled clinical trials to confirm the efficacy of apatinib on advanced HCC.

## MATERIALS AND METHODS

### Patients

From September 14, 2015 to October 17, 2016, 22 patients with advanced HCC received apatinib treatment in the Tianjin Medical University Cancer Institute and Hospital. All patients satisfied the following criteria: Barcelona Clinic Liver Cancer (BCLC) stage C; 18 years of age or older; Eastern Cooperative Oncology Group (ECOG) score 0-2; Child-Pugh classification of liver function A-B; more than 4 weeks of medication time; complete imaging data; followed the imaging requirements of the Response Evaluation Criteria in Solid Tumors (RECIST) 1.1 [26]; and consented to receive apatinib treatment. Patients were diagnosed with HCC by two imaging modalities, or postoperative pathology, or biopsy.

### Therapeutic procedures

The recommended apatinib dose reported for advanced gastric adenocarcinoma or gastroesophageal junction adenocarcinoma is 850 mg/day orally [8]. However, considering that liver cirrhosis and hepatic insufficiency are common among our selected HCC patients, the first 6 patients were given apatinib at 500 mg/day taken 2h after dinner. Unfortunately, none of these patients tolerated the serious adverse events that ensued. Thereafter, we implemented a starting dose of 250 mg/day in subsequent patients. Treatment would be interrupted or terminated under the following conditions: serious adverse events, death of patients, or voluntarily giving up. If grade 3 or 4 adverse events occurred during apatinib treatment, we would first suspend it for 1-2 weeks to alleviate the side effects; then, apatinib would be continued. Treatment interruptions and up to one dose reductions (250 mg every 2 days) were permitted in case of drug-related adverse events. If further dose reductions were required, patients were withdrawn from the study.

### Outcomes and assessments

Blood counts, liver and renal function, and hepatic tumor biomarker tests were performed monthly. MRI or CT scans of measurable lesions were assessed at screening and every 8 weeks thereafter. RECIST version 1.1 was used to assess tumor responses. Disease control was defined as CR, PR, SD, and PD. TTP, ORR, DCR, and OS were used for evaluation of clinical endpoints.

### Statistical analysis

Statistical analyses were performed using SPSS statistical package version 18.0 (SPSS, Chicago, USA). Kaplan-Meier plots were used to estimate the time of HCC progression and survival time. Cox proportional hazard analysis was done to assess the effect of important baseline characteristics on overall survival. HR and 95% CIs were estimated using a non-parametric log-rank test. P < 0.05 was considered as statistically significant.

## SUPPLEMENTARY MATERIALS FIGURES





## Abbreviations

HCC: hepatocellular carcinoma
BCLC: Barcelona Clinic Liver Cancer
CR: complete response
PR: partial response
SD: stable disease
PD: progressive disease
TTP: time to disease progression
OS: overall survival
ORR: objective response rate
DCR: disease control rate
