# Supplementary material for: Apatinib is effective for treatment of advanced hepatocellular carcinoma
Source: Oncotarget. 2017 Nov 6;8(62):105596–605. doi: 10.18632/oncotarget.22337 (PMC5739662; doi:10.18632/oncotarget.22337)
Supplement: Supplementary file 1 [file oncotarget-08-105596-s001.pdf]

Apatinib is effective for treatment of advanced hepatocellular carcinoma

SUPPLEMENTARY MATERIALS

Supplementary Figures: Typical imaging changes of 9 PR patients.

See Supplementary File 1
