# Supplementary material for: Apatinib is effective for treatment of advanced hepatocellular carcinoma
Source: Oncotarget. 2017 Nov 6;8(62):105596–605. doi: 10.18632/oncotarget.22337 (PMC5739662; doi:10.18632/oncotarget.22337)
Supplement: Supplementary file 2 [file oncotarget-08-105596-s002.docx]

**Apatinib is Effective for Treatment of Advanced Hepatocellular Carcinoma**

Yinlong Kong^1^, Lin Sun^1^, Zhenyu Hou^1^, Yongqiang Zhang^1^, Ping Chen^1^, Yunlong Cui^1^, Xiaolin Zhu^1^, Tianqiang Song^1^, Qiang Li^1^, Huikai Li^1^, Ti Zhang^1^ and Lunxiu Qin^2,3^

^1^ Tianjin Medical University Cancer Institute and Hospital, Key laboratory of Cancer Prevention and Therapy, National Clinical Research Center for Cancer, Tianjin 300060, China

^2^ Department of General Surgery, Huashan Hospital & Cancer Metastasis Institute, Fudan University, Shanghai 200040, China

^3^ Cancer Research Center, Institutes of Biomedical Science, Fudan University, Shanghai 200032, China

Correspondence to:

Ti Zhang, E-mail: [zhangti@tjmuch.com](mailto:zhangti@tjmuch.com)

Huikai Li, E-mail: [tjchlhk@126.com](mailto:tjchlhk@126.com)

Lunxiu Qin, Email: [qinlx99@vip.163.com](mailto:qinlx99@vip.163.com)

**Supplementary Data:**Typical imaging changes of 9 PR patients

Case 1 JLY (PR)

**Both of tumor in liver (red arrow) and emblus in vena cava (yellow arrow) shrinked significantly , and AFP decreased dramatically**

Case 2 XSW (PR)

**Intrahepatic metastatic HCC tumors (yellow circled) disappeared and the metastatic retroperitoneal lymph node (blue circled) shrinked dramatically after the treatment, and AFP decreased to normal level**

Case 4 LGW (PR)

**Both of metastatic infraclavicular (top) and retroperitoneal lymph node (bottom) shrinked significantly, and AFP decreased dramatically**

Case 8 LJY (PR)

**Metastatic retroperitoneal lymph node (arrow) shrinked significantly , and AFP decreased dramatically after two months treatment.**

Case 11 YH (PR)

**Tumor in liver (red circled) and portal vein tumor thrombosis (yellow arrow) shrinked significantly with decreased AFP after three months treatment**

Case 13 YZM (PR)

**Both of tumor size and activity in liver (red arrow) and emblus in portal vein (yellow arrow) decreased dramatically after three months treatment**

Case 15 ZSL (PR)

**Tumor in liver, abdominal cavity and lung shrinked significantly , and AFP decreased to normal level after three months treatment**

Case 16 BDX (PR)

**Tumor in liver (yellow arrow) shrinked significantly with decreased AFP after two months apatinib treatment; tumor necrosis (red arrow) was also found**

Case 17 ZXY (PR)

**Metastatic tumors in lung shrinked significantly with decreased AFP after three months treatment of apatinib**
